# Superconductivity in Nb: Impact of Temperature, Dimensionality and Cooper-Pairing

**DOI:** 10.3390/nano14030254

**Published:** 2024-01-24

**Authors:** Uriel Allan Aceves Rodriguez, Filipe Souza Mendes Guimarães, Samir Lounis

**Affiliations:** 1 Peter Grünberg Institut & Institute for Advanced Simulation, Forschungszentrum Jülich & JARA, D-52425 Jülich, Germany; u.aceves@fz-juelich.de; 2 Faculty of Physics & CENIDE, University of Duisburg-Essen, D-47053 Duisburg, Germany; 3 Jülich Supercomputing Centre, Forschungszentrum Jülich & JARA, D-52425 Jülich, Germany; f.guimaraes@fz-juelich.de

**Keywords:** superconductivity, Bogoliubov-de Gennes formalism, bulk & films, superconducting gap, tight-binding simulations

## Abstract

The ability to realistically simulate the electronic structure of superconducting materials is important to understand and predict various properties emerging in both the superconducting topological and spintronics realms. We introduce a tight-binding implementation of the Bogoliubov–de Gennes method, parameterized from density functional theory, which we utilize to explore the bulk and thin films of Nb, known to host a significant superconducting gap. The latter is useful for various applications such as the exploration of trivial and topological in-gap states. Here, we focus on the simulation’s aspects of superconductivity and study the impact of temperature, Cooper-pair coupling and dimensionality on the value of the superconducting pairing interactions and gaps.

## 1. Introduction

Superconductivity is a basic fundamental phenomenon in solid-state physics. Although being discovered more than a century ago, it still generates a plethora of research activities. Recently, its importance flourished again in the context of topological superconductivity [1,2,3], which is an essential field of research for the realization of quantum computing. Certainly, the intricate interplay of standard spintronics and quantum information concepts can give rise to unforeseen breakthroughs in basic research and in future technologies for storing, computing and transmitting information. This calls for the development of theoretical frameworks based on a realistic description of the electronic structure of superconducting materials interfaced with magnetic systems in order to understand and predict various related phenomena.

Interesting topics emerged in superconducting spintronics [4,5,6,7,8], where the interplay of Cooper pairs and other electronic degrees of freedom (such as spin, charge and orbital) give rise to fascinating phenomena, ranging from supercurrents, triplet superconductivity, and to supercurrent-driven spin transfer and spin-orbit torques. Simultaneously, the potential design of topological superconductivity and the creation of Majorana states [9,10,11] are driving a lot of research in the context of topological qubits [12].

The microscopic theory to describe conventional superconductivity goes back to the work of John Bardeen, Leon Cooper, and Robert Schrieffer (BCS) [13]. In this context, the Bogoliubov–de Gennes (BdG) method [14,15,16,17] is an elegant mean-field approximation that relies upon Bogoliubov–Valatin transformations that take the Hamiltonian from a particle space into a particle-hole one—a framework that has been frequently used, see for example Refs. [18,19,20,21,22,23,24,25,26,27,28]. Nowadays, several methods based on a realistic description of the electronic structure of materials are capable of treating fundamental aspects related to superconductivity. For instance, powerful theoretical frameworks have been proposed and utilized within density functional theory (DFT) combined with the BdG formalism [29,30,31,32,33,34], or by proposing an extension of DFT to account for superconductivity [35,36,37,38]. Tight-binding schemes are very appealing since they allow a versatile description of superconductivity-related physics with a potential control of various levels of approximations, which could enable the treatment of problems not attainable with conventional DFT.

Among the superconducting materials, Nb stands out and is often utilized as a key component to investigate superconductivity-related physics [39,40]. The demonstration of the ability of growing clean Nb(110) surfaces [41] promoted the latter to be the ideal substrate to prospect Yu-Shiba-Rusinov in-gap states [42,43,44] and the topological Majorana states [9,10,11] as explored in atoms [30,45,46,47], at the edges of magnetic wires [25,27,28,48], or films [49,50,51,52].

The goal of this article is to introduce our recently developed method that allows the treatment of superconductivity on the basis of a tight-binding approach as implemented in TIme-dependent Transport and Angular momentum in Nanostructures (TITAN) [53,54,55,56]. The framework enables a realistic description of the electronic structure, with parameters obtained from DFT [57]. Examples of applications of TITAN ranges from dynamical magnetic responses [58], dynamical transport and torques [54,56], magnetic damping [55], as well as ultrafast magnetization dynamics triggered by laser pulses [59,60]. In the context of superconductivity, the method was already introduced in Ref. [61], with a focus on magnetic exchange interactions at superconducting-magnetic interfaces. Here, we pay attention to Nb in bulk and thin films with the aim of highlighting fundamental aspects in the simulation procedure of superconducting properties. Aspects related to the choice of the electron–phonon coupling to open the appropriate band gap are discussed together with the impact of temperature and self-consistency on the results of the simulations. We address in particular systematic repeated simulations to find the coupling that results in the experimentally observed value of the superconducting gap. These simulations consume a lot of computing time since the gaps to be resolved are of the order of meV (or smaller), which require a large number of *k*-points and simulations to properly resolve the underlying electronic band-structure. We propose a promising numerical path to improve the search time to identify the optimal electron–phonon coupling. Furthermore, we explore how dimensionality affects superconductivity in the particular case of Nb films, which can be utilized to tune the superconducting gap for different applications in modern investigations of superconductivity-related problems. For instance, we predict that a single Nb monolayer can be superconducting similarly to the bulk phase.

This article is organized as follows. We first introduce the method in Section 2, which is based on multi-orbital tight-binding theory. Then, we present in Section 3 the results of our simulations in Nb bulk, (001) and (110) monolayers, which are followed by Nb(110) thin films of different thicknesses. Finally, we present our concluding remarks in Section 4.

## 2. Tight-Binding and the Bogoliubov–de Gennes Method

To simulate the superconducting properties of Nb across dimensions, we introduce here the tight-binding methodology together with the BdG method that we utilized, as implemented in our code TITAN [53,55,56,59,60,61]. The hopping parameters were obtained from first-principles calculations [57]. More details on the aspects related to superconductivity are elaborated in Ref. [61].

The fundamental tight-binding Hamiltonian reads:

(1) $$\begin{array}{c} H_{S}=\frac{1}{N}\left\{\sum_{\alpha \beta ,\sigma \eta ,\mu \nu ,k}H_{\alpha \beta ,\sigma \eta }^{\mu \nu }\left(k\right)c_{\alpha \mu \sigma }^{†}\left(k\right)c_{\beta \nu \eta }\left(k\right)-\sum_{\alpha ,\mu \nu ,kk^{\prime }}\lambda_{\alpha \mu }c_{\alpha \mu ↑}^{†}\left(k\right)c_{\alpha \mu ↓}^{†}\left(-k\right)c_{\alpha \mu ↓}\left(-k^{\prime }\right)c_{\alpha \mu ↑}\left(k^{\prime }\right)\right\}, \end{array}$$

where $c_{\alpha \mu \sigma }^{†}\left(k\right)$ and $c_{\beta \nu \eta }\left(k\right)$ consist of the creation and annihilation operators associated to electrons having a wave vector k and spin σ in the orbitals μ and spin η in the orbitals ν, respectively. Items α and β are specific atoms in the bulk unit cell, or layers in the film geometry. Item k is a reciprocal vector, while *N* is the number of wave vectors in the three-dimensional (for bulk) or two-dimensional (for films) Brillouin zone. To enable the formation of Cooper pairs, which ultimately gives rise to superconductivity, we introduce $\lambda_{\alpha \mu }$ in the second term of the previous equation, as induced by the electron-phonon coupling. λ is a pairing interaction term that enables the electron-hole coupling responsible for the opening of the superconducting gap as described in the BCS theory. In our scheme, it retains an orbital-dependent nature.

In the mean-field approximation, Equation (1) simplifies to

(2) $$\begin{array}{cc} H_{S}^{\mathrm{MF}}= & \frac{1}{N}\sum_{k}\left\{\sum_{\alpha \beta ,\sigma \eta ,\mu \nu }H_{\alpha \beta ,\sigma \eta }^{\mu \nu }\left(k\right)c_{\alpha \mu \sigma }^{†}\left(k\right)c_{\beta \nu \eta }\left(k\right)\right.\\ & -\left.\sum_{\alpha ,\mu }\left(D_{\alpha \mu }^{*}c_{\alpha \mu ↓}\left(-k\right)c_{\alpha \mu ↑}\left(k\right)+D_{\alpha \mu }c_{\alpha \mu ↑}^{†}\left(k\right)c_{\alpha \mu ↓}^{†}\left(-k\right)\right)\right\}, \end{array}$$

with

(3) $$D_{\alpha \mu }=\lambda_{\alpha \mu }\frac{1}{N}\sum_{k}\langle c_{\alpha \mu ↓}\left(-k\right)c_{\alpha \mu ↑}\left(k\right)\rangle ,\;D_{\alpha \mu }^{*}=\lambda_{\alpha \mu }\frac{1}{N}\sum_{k}\langle c_{\alpha \mu ↑}^{†}\left(k\right)c_{\alpha \mu ↓}^{†}\left(-k\right)\rangle ,$$

$D_{\alpha \mu }$ is known as the superconducting pair interaction, which corresponds to the energy required to scatter Cooper pairs [62].

The term $H_{\alpha \beta ,\sigma \eta }^{\mu \nu }\left(k\right)$ corresponds to the regular Hamiltonian where superconductivity is not accounted for:

(4) $$H_{\alpha \beta ,\sigma \eta }^{\mu \nu }\left(k\right)=H_{\alpha \beta }^{0\mu \nu }\left(k\right)\sigma^{0}+\sigma \cdot {\hat{e}}_{\alpha }B_{\alpha }^{[\mathrm{xc}]\mu \nu }\left(k\right)\delta_{\alpha \beta }+\sigma \cdot B_{\alpha }^{[\mathrm{soc}]\mu \nu }\left(k\right)\delta_{\alpha \beta },$$

with $H_{\alpha \beta }^{0\mu \nu }$ being the spin-independent tight-binding term, the second term is responsible for the intra-atomic exchange interaction (originating from a Hubbard-like contribution [54,56]), which can lead to magnetism, and the third term corresponds to the spin-orbit interaction.

The BdG methodology consists in utilizing the Bogoliubov–Valatin transformation [17] to rewrite Equation (2) in the electron-hole representation instead of the electron one, which leads to the BdG Hamiltonian:

(5) $$H_{\mathrm{BdG}}^{\alpha \beta ,\mu \nu }\left(k\right)=\left(\begin{array}{cccc} H_{\alpha \beta ,↑↑}^{\mu \nu }\left(k\right)-E_{F} & H_{\alpha \beta ,↑↓}^{\mu \nu }\left(k\right) & 0 & -D_{\alpha \mu }I\\ H_{\alpha \beta ,↓↑}^{\mu \nu }\left(k\right) & H_{\alpha \beta ,↓↓}^{\mu \nu }\left(k\right)-E_{F} & D_{\alpha \mu }I & 0\\ 0 & D_{\alpha \mu }^{*}I & -H_{\alpha \beta ,↑↑}^{\mu \nu *}\left(-k\right)+E_{F} & -H_{\alpha \beta ,↑↓}^{\mu \nu *}\left(-k\right)\\ -D_{\alpha \mu }^{*}I & 0 & -H_{\alpha \beta ,↓↑}^{\mu \nu *}\left(-k\right) & -H_{\alpha \beta ,↓↓}^{\mu \nu *}\left(-k\right)+E_{F} \end{array}\right),$$

with $E_{F}$ being the Fermi energy while the associated eigenvalue problem to solve reads:

(6) $$\sum_{\beta \mu }H_{\mathrm{BdG}}^{\alpha \beta ,\mu \nu }\left(k\right)\phi_{\beta \mu }\left(k\right)=E_{n}\left(k\right)\phi_{\alpha \nu }\left(k\right).$$

The transformation is canonical and enabled by rewriting

(7) $$c_{\alpha \mu \sigma }\left(k\right)=\sum_{n}^{\prime }u_{\alpha \sigma }^{n}\left(k\right)\gamma_{n}+v_{\alpha \sigma }^{n*}\left(k\right)\gamma_{n}^{†},\;c_{\alpha \mu \sigma }^{†}\left(k\right)=\sum_{n}^{\prime }u_{\alpha \sigma }^{n*}\left(k\right)\gamma_{n}^{†}+v_{\alpha \sigma }^{n}\left(k\right)\gamma_{n}^{†},$$

with

(8) $$\left\{\gamma_{n},\gamma_{m}\right\}=\left\{\gamma_{n}^{†},\gamma_{m}^{†}\right\}=0,\;\left\{\gamma_{n}^{†},\gamma_{m}\right\}=\delta_{nm},$$

where the prime indicates that the sums run only over the states with positive energy [15,17]. This restriction in the sum is carried out to counteract the doubling of the degrees of freedom originated from the change of basis.

The eigenvector of Equation (6) is

(9) $$\phi_{i\nu }\left(k\right)=\left(\begin{array}{c} u_{\alpha \nu ↑}\left(k\right)\\ u_{\alpha \nu ↓}\left(k\right)\\ v_{\alpha \nu ↑}\left(k\right)\\ v_{\alpha \nu ↓}\left(k\right) \end{array}\right).$$

Our numerical procedure to evaluate the various properties of a given material is based on the self-consistency of the charge density, which is also pursued once the electron–phonon coupling is included. In practice, we start the simulations considering the normal metallic phase; then, we incorporate the Cooper pairing and repeat the computational runs until convergence.

## 3. Results

In this section, we explore the results of simulations conducted for various Nb structures. Our choice of Nb is motivated by its relatively large superconducting gap, which facilitates the exploration of in-gap states. Simulating low temperature phenomena requires finer computational meshes; thus, for the first investigations, we choose high temperatures and large values for the superconducting coupling λ. We start with bulk Nb, where we recover a superconducting gap opening in the electronic structure around the Fermi energy. Then, we explore the case of thin films down to the single monolayer along the [001] or [110] directions. We monitor how the superconducting gap is affected by the self-consistency of the electronic structure calculations.

### 3.1. Bulk Superconducting Nb

Nb has a superconducting critical temperature of $T_{c}=9.3\;K$ [63], which leads to a significant superconducting gap 2Δ=3.8 meV. A rough approximation relates $T_{c}$ with the size of the gap—at T = 0 K—through [62]

(10) $$\Delta \left(T=0\right)=1.764k_{b}T_{c}.$$

**Figure 1:**
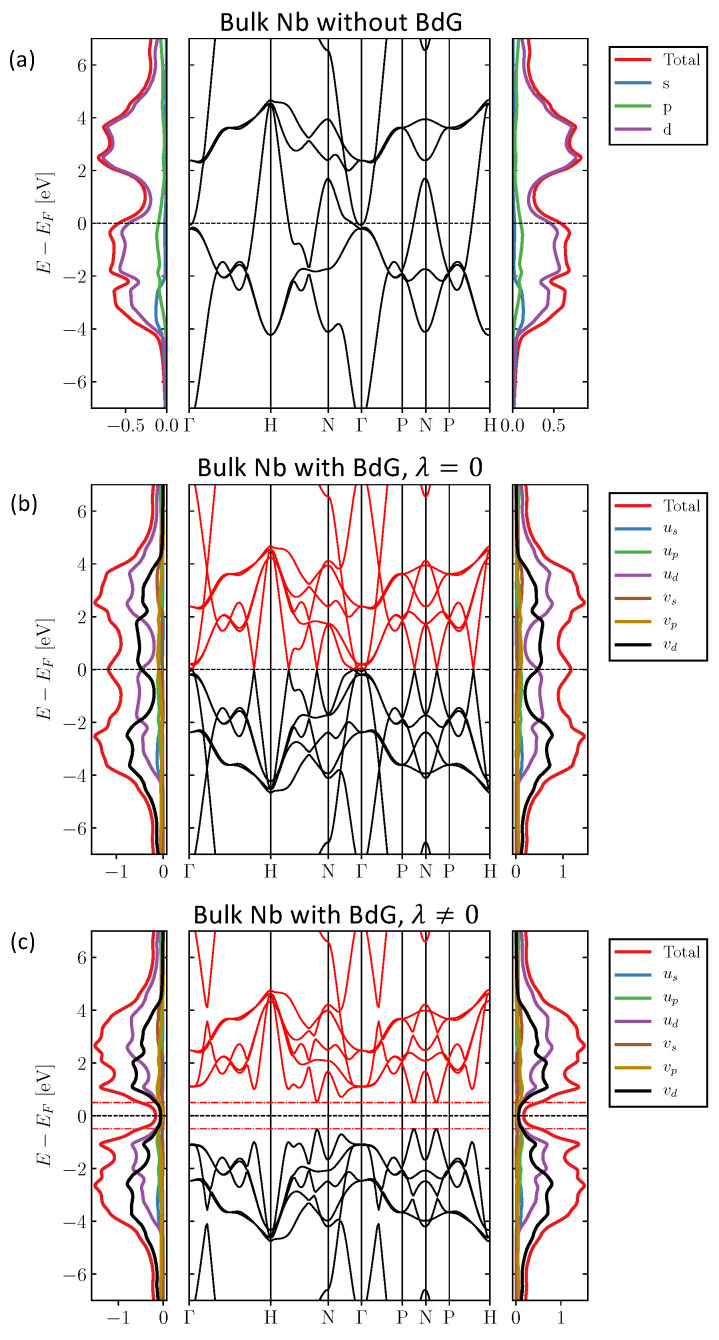
Electronic structure of bulk Nb with and without superconductivity. Three cases are illustrated: (**a**) without the BdG formalism only the electron part is shown; (**b**) with the BdG formalism but setting the Cooper pair coupling λ to zero; (**c**) with BdG and λ=5.44 eV in all orbitals. The red colored line evinces the location of the gap as predicted by the self-consistent value Δ=0.497 eV and thermal broadening 43 meV. The band structure is shown in the middle. To the right (left), the DOS of the majority (minority) spin channel is presented. The legend box depicts the colors used to signal the total, the band resolved DOS and their electron versus hole parts, i.e., the contributions from, respectively, the *u* and *v* components of the eigenvectors.

Large values of Δ are desirable when investigating states that appear inside the gap. In our simulation procedure, we start with bulk Nb at high temperatures ($k_{b}T=43\;meV$). For a reference, this is higher than room temperature, which is at 26 meV, but nevertheless, considerably lower than the melting point of Nb 237 meV. Nb has a body-centered cubic crystal structure. We make use of the Slater–Koster (SK) parameters from Ref. [57] and the spin-orbit coupling strength comes from Ref. [64]. In Figure 1a, we show the density of states of both spin channels (left and right panels), and in the middle the band structure of bulk Nb. The occupation of electronic orbitals is $\langle n_{s}\rangle =0.70,\;\langle n_{p}\rangle =0.68,\;\langle n_{d}\rangle =3.61.$ The density of states (DOS) and the band structure agree with the results of Ref. [57].

Repeating the calculations by solving the BdG equations but setting λ=0 on all orbitals, we naturally recover the metallic band structure shown in Figure 1b, where both the electron and hole parts of the band-structure are illustrated. Both parts are symmetric with respect to the Fermi level. This holds true for the DOS as well. We expect that by allowing for finite coupling λ, degeneracies occurring at crossing points will be lifted.

In Section 3.2, we describe a rather optimized search method to tune this parameter. From our explorations, we found that: (i) large values of λ do not always mean large gaps, and (ii) the size of the gap is not a linear function of λ. From tabulated values of the electron–phonon $\lambda_{e-p}$ coupling constant [65], we can evaluate the coupling parameter λ utilizing the following relation

(11) $$\lambda =\lambda_{e-p}D\left(E_{F}\right),$$

where $D(E_{F})$ is the density of states at the Fermi energy. One can use the McMillan formula [66,67] with the electron–phonon coupling evaluated via density functional perturbation theory [68,69]. For example, for bulk Nb with the data from Ref. [70] we obtain λ≈1.3 eV. In Ref. [65], the electron–phonon coupling constant is calculated for slabs of Nb(100) and Nb(110) of 3, 6, 9, 12, and 15 layers. For the Nb(110), the results for the system of nine layers—the slab size closer to the one we will study later—lead to coupling parameters of λ≈5.28 eV at the surfaces, and λ≈1.3 eV in the middle layer. When plotting the curve associated with Δ vs. λ, we obtain a quadratic function, in agreement with Ref. [71], where the self-consistent Korringa–Kohn–Rostoker method is used. There, the authors found that in order to obtain Δ≈1 meV, the coupling parameter must be λ≈1.1 eV for bulk Nb.

We note that although not-trivial, the electron–phonon coupling can be extracted via state-of-the-art experiments, for example via inelastic neutron scattering experiments [72], angle-resolved photoemission spectroscopy (ARPES) [73], time-resolved ARPES [74] and inelastic scanning tunneling microscopy [75].

As a first example of our investigations, we set $\lambda_{\mu }=5.44\;eV$. The occupancies are $\langle n_{s}\rangle =0.70,\;\langle n_{p}\rangle =0.67,\;\langle n_{d}\rangle =3.63$, which experience a minor change in the order of $10^{-2}$ with respect to the case where λ is set to zero. The self-consistent value of the superconducting gap parameter is Δ=0.497 eV, which is obviously gigantic. The resulting band structure and LDOS plots are illustrated in Figure 1c. The red dotted lines delimit the range E∈[−Δ,Δ]. As a sanity check, it is reassuring that the gap in the band structure matches the self-consistent output value of Δ. The density of states does not show a perfect gap, but decays at the gap region. This effect is due to the thermal broadening induced artificially, which is in this case set to 43 meV.

In Section 3.2 and Section 3.4, we will systematically explore the impact of temperature and coupling strengths, searching for an optimal λ that yields the experimental value of Δ.

### 3.2. Superconducting Gap Tuning: Single Bcc (001) Nb Monolayer

Explorations to find the appropriate coupling λ that results in the desired gap parameter Δ involve systematic repeated simulations with different parameters. In this section, we show the typical curves for Δ as a function of the temperature (via the broadening parameter) and the Cooper pair coupling parameter. To exemplify, we use a 2D system, namely a (001) monolayer of bcc Nb. As in our previous examples, we set the same λ for all orbitals, with eight values between 4 and 9 eV. We consider forty different temperature points between 300 K and 14,000 K. Simulations at lower temperatures are computationally highly expensive since they require an increased number of k-points to be able to capture the narrow peaky structures in the Brillouin zone with lower broadening. All the simulations in this section use the same number of k-points, ≈$2\times 10^{6}$.

**Figure 2:**
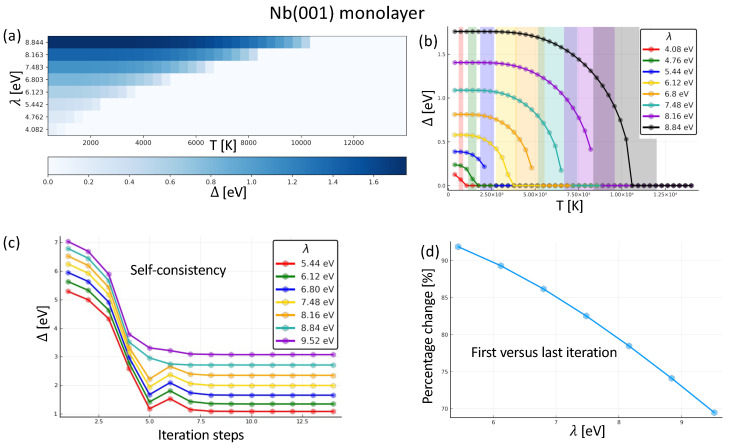
Dependence of the superconducting gap of Nb(001) monolayer on various parameters. (**a**) Heat map of the superconducting gap as function of the coupling λ and the thermal broadening parameter. All curves drop to zero after reaching their corresponding critical temperature. (**b**) Another version of (**a**) in order to better follow how the superconducting gap evolves against temperature. The bands in color reflect an rough extrapolation to the critical temperature. (**c**) Evolution of the superconducting gap across the self-consistency for several coupling strengths λ. (**d**) Relative change of the superconducting gap when comparing the values obtained after one single iteration to those after self-consistency ($\left(\Delta_{\mathrm{initial}}-\Delta_{\mathrm{final}}\right)/\Delta_{\mathrm{initial}}$) for different values of λ.

Figure 2a displays a heatmap with the results of the gap parameter for the different temperatures and couplings. We can see that the gap parameter drops to zero after reaching the corresponding critical temperature for every line. Directing our attention for fixed temperatures (i.e., the “columns” in the figure), it is clear that there is also a critical $\lambda_{c}$ below which Δ is zero. Throughout the heatmap, Δ ranges from 0.0 to 1.76 eV. We can analyze the same data from a different perspective, as shown in Figure 2b. There, we see that the Δ vs. *T* curve has indeed the shape predicted by the BCS theory. For sufficiently low temperatures, Δ approaches a plateau. Around the critical temperature $T_{c}$, the self-consistency procedure may become unstable and difficult to achieve a precise value, which lead some curves to miss points in that range. We can proceed to an extrapolation to evaluate the missing points on the basis of the asymptotic behavior of Δ around the critical temperature [62]

(12) $$\Delta \left(T\right)\approx 1.74\Delta \left(0\right)\sqrt{1-\frac{T}{T_{c}}}.$$

We used the precedent equation to go from the data in Figure 2b to the one in Figure 2a. The BCS theory predicts that, at zero Kelvin, the relation between Δ and $T_{c}$ is the one in Equation (10). Experimentally the reported values range from $2\Delta =3.2k_{b}T_{c}$ to $2\Delta =4.6k_{b}T_{c}$, we signaled this range with the colored bands in Figure 2b. This plot establishes the aforementioned statement regarding the non-linearity of Δ with respect to λ.

For a wide range of simulations, it is desirable and recommendable to place the superconducting system well below $T_{c}$, at a point in the plateau of the Δ vs. *T* curve.

### 3.3. Renormalization after Self-Consistency: Single Bcc (001) Nb Monolayer

In regular simulations of superconductivity, the electronic structure is not allowed to converge self-consistently. This assumes that the electronic structure is not significantly altered in comparison to the case, where instead, one proceeds to self-consistency. The importance of self-consistency was highlighted in Ref. [76]. In this subsection, we address this aspect of simulation of superconducting materials by comparing the size of the gap obtained after one iteration (starting from the converged electronic structure of the metallic phase) with respect to the one obtained after full self-consistency. In Figure 2c, we single out the value of the superconducting gap parameter through several simulations with different coupling parameters at a temperature of 43 meV. Our algorithm, based on the Powell method [77] can halt the simulations when no improvement is detected in the last five steps. All shown cases converged without a problem.

In Figure 2d we portray the percentage change of the gap parameter ($\left(\Delta_{\mathrm{initial}}-\Delta_{\mathrm{final}}\right)/\Delta_{\mathrm{initial}}$) between the first- and last-iteration calculated Δ for different λ. We note that the change is bigger for smaller values of λ.

So far, we have been discussing the numerics of the simulations assuming both large superconducting gaps and large electronic temperatures. In Section 3.4, we drop *T* to realistic values associated with a superconducting Nb layer. Additionally, we explore the impact on the orbital-dependent gaps $\Delta_{\mu }$.

### 3.4. Superconducting Nb (110) Monolayer

In this subsection, we consider the cryogenic regime, which calls for computationally heavier simulations. As we hinted in previous subsections, sinking the temperature forces us to increase the number of k-points for each simulation to be able to capture the narrow states. Since these calculations will be more intensive, we will leave aside the testing structures we were using previously—bulk Nb and Nb (100) monolayers—and move to our main interest, which is Nb (110) films, heavily explored in various recent experiments.

**Figure 3:**
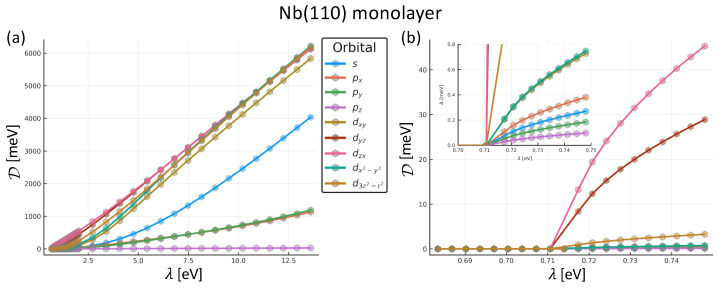
Orbital-resolved superconducting pairing interaction for Nb(110) monolayer. (**a**) $D_{\mu }$ grows considerably for all the orbital except $p_{z}$. (**b**) Two close ups to the region where the material transitions from the non-superconducting to the superconducting phase. Simulations performed at 4.96 K.

We assume a thermal broadening of approximately 4.96 K, shallow enough to be in the plateau of the Δ vs. *T* curve for all the λ we used. We consider a non-uniform grid with 57 points for λ, ranging from 0.68 eV to 13.6 eV, with a higher resolution for lower energies. In this subsection, we pay special attention to the orbital-dependent superconducting pairing interaction $D_{\mu }$, which ultimately is responsible for the opening of the superconducting gap Δ. In Figure 3a, we display $D_{\mu }$ for the entire range of considered λ values. Obviously, some orbitals are more responsive to the coupling λ. This is affected by the strength of the latter coupling. We notice an increment in D as we enlarge the coupling strength.

In Figure 3b, we focus only on the low energy range λ∈[0.5 eV,0.75 eV], where the growth of $D_{\mu }\left(\lambda \right)$ is more controlled. In the inset, we also restrict the values on the *y*-axis from 0 to 0.8 eV to resolve better the smaller superconducting pairing interaction for some of the orbitals. We observe that the $p_{z}$ component is still quite small; this is a consequence of the shape of this orbital and the geometry of the monolayer. The orientation of the $p_{z}$ orbital is out of the plane, and this means its in-plane component is zero, and it is hard for superconducting electrons in this orbital to interact with others.

The orbital-dependent superconducting pairing interaction λ is responsible for the opening of the superconducting gap, once plugged in Equation (5). In contrast to models with a single orbital, the final gap in a realistic multi-orbital case results from non-trivial interferences and combinations of different contributions.

The gap is opened around the Fermi surface when the *u* and *v* states mix due to the off-diagonal components of the Hamiltonian; this means that the gap must come from the bands that cross $E_{F}$. Once we profile these bands, we can execute any searching algorithm—e.g., binary search, or nested intervals methods—until we find an appropriate λ. As a rule of thumb, each significant figure in Δ requires two on λ; in other words, having Δ up to three significant digits typically requires knowing λ to six. Refining can be very expensive. In our case, λ=1.37 eV induces Δ=1.4 meV.

In the next subsection, we explore the case of thin films of Nb (110) at 4.96 K and analyse the evolution of the superconducting gap parameter across the material.

### 3.5. Superconducting Slab

It is difficult to predict if the coupling strength λ that works well for one given system still opens a reasonable gap for a different one. We started with the value found in the previous section, λ=1.37 eV, which opened a gap Δ=1.4 meV for a single monolayer. It turns out, however, that as soon as the Nb film thickness is larger than five layers, a large coupling is required to open a gap. In the remained study, we assume λ=2.72 eV at a temperature of 4.96 K.

**Figure 4:**
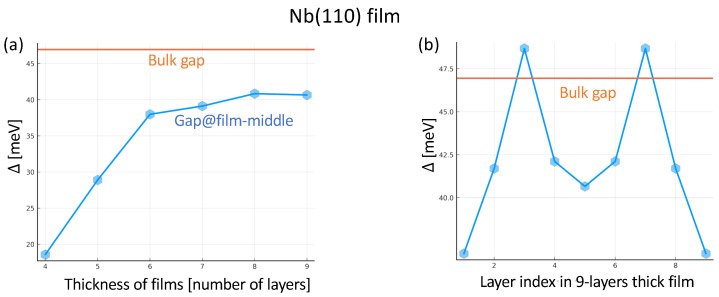
Superconducting gap for Nb(110) films. (**a**) Superconducting gap at the centers of Nb(110) films of different thicknesses. (**b**) Layer-resolved superconducting gap for a nine-layer-thick Nb(110) film. The straight orange line represents the gap (46.9 meV) associated to bulk Nb assuming λ=2.72 eV and a temperature of 4.96 K.

In Figure 4a, we show the magnitude of the gap in the central layer of the films as a function of the slab thickness. The orange line corresponds to the gap parameter of the bulk Nb for λ=2.72 eV at the same temperature. One notices that the thicker the film gets, the closer the gap is to the one obtained for the bulk, a behavior comparable to what has been reported in Ref. [78].

The superconducting gap is not homogeneous across the Nb films. An example is shown in Figure 4b for a film with a thickness of nine layers. The gap in the middle of the film is larger than at the surface, and it seems to oscillate as a function of position, as we would expect for wave-like particles in a confined system. A lower gap at the surface is expected since the electrons there have fewer opportunities to couple in Cooper-pairs due to their lack of neighbors from one side. We must also consider surface effects; for example, we have around 1.5 electrons less than the inner parts. Electrons move from the surfaces to the middle layers. Interestingly, some of the layers in the film can have a larger gap than the bulk one. This indicates that the dimensionality of a material can be utilized to control the superconducting gap due to confinement effects.

## 4. Conclusions

After briefly introducing the tight-binding method that we developed to tackle superconductivity within the Bogoliubov–de Gennes formalism, we presented the results of our simulations on a material that is currently heavily utilized to explore the physics of superconductivity. We addressed the case of bulk and thin films of Nb along different directions. A major issue in this field from the computational point of view is to choose the right Cooper pair coupling that opens the desired superconducting gap since the calculations are extremely expensive to resolve gaps of a few meV or even smaller. We analyzed how the superconducting gap correlates with both the Cooper pair coupling and temperature, and explored the impact of self-consistency on the emerging orbital-dependent superconducting pairing interaction. In the thin film geometry, we unveiled the possibility of engineering the magnitude of the gap via confinement effects. Indeed, the superconducting gap is found to be layer-dependent, either smaller or larger than the values found in the bulk phase.

## Data Availability

All data needed to evaluate the conclusions of the paper are present in the paper. Additional data related to this paper may be requested from the authors.
